# Comparative effect of stenting plus medical therapy vs medical therapy alone on the risk of stroke and death in patients with symptomatic intracranial stenosis: a systematic review and meta-analysis

**DOI:** 10.1007/s00415-022-11429-9

**Published:** 2022-10-27

**Authors:** Xin Wu, Jiaxuan Li, Shixin Wang, Yu Zou, Liyan Tang, Zhouqing Chen, Wei Zhang, Zhong Wang

**Affiliations:** 1 Department of Neurosurgery, Suzhou Ninth People’s Hospital, Suzhou, 215200 Jiangsu Province China; 2 Department of Neurosurgery & Brain and Nerve Research Laboratory, The First Affiliated Hospital of Soochow University, 188 Shizi Street, Suzhou, 215006 Jiangsu Province China

**Keywords:** Stenting therapy, Symptomatic intracranial stenosis, Ischaemia, Stroke, Meta-analysis

## Abstract

**Background:**

Recently, several randomized controlled trials (RCTs) of stenting plus medical therapy versus medical therapy alone have been successfully conducted for the treatment of patients with symptomatic intracranial stenosis. This study aimed to evaluate differences between these two therapies in the risk of stroke and death.

**Methods:**

MEDLINE, EMBASE, the Cochrane Library, and ClinicalTrials.gov were systematically searched to identify relevant studies published before August 24, 2022. Review Manager 5.3 software was used to assess the data. The risk ratio (RR) was analysed and calculated with a random effect model or a fixed effects model.

**Results:**

We pooled 921 participants from three RCTs. Compared to the medical therapy alone group, the stenting plus medical therapy group had a higher risk of 30-day death or stroke (RR = 2.69 [1.64–4.41], *P* < 0.0001, *I*^2^ = 0%). When the follow-up period exceeded 1 year (≥ 1 year), there was no significant difference in the risk of stroke or death between these two groups. The subgroup analysis showed that if the time from stroke onset to implantation was extended, additional stenting would have no effect on the risk of stroke or death, whether within 30 days or within 1 year (*P* = 0.16 and 0.78).

**Conclusion:**

Medical therapy alone has a lower risk of stroke and death in the short term than stenting plus medical therapy, while no difference exists in the long term. More studies are still needed to further explore the precision strategy of stent implantation for symptomatic intracranial stenosis patients.

**Supplementary Information:**

The online version contains supplementary material available at 10.1007/s00415-022-11429-9.

## Introduction

Stroke occurs in over 12.2 million patients each year [1] and is the leading cause of mortality and disability worldwide [2, 3]. Intracranial stenosis is a common vascular disease in stroke patients and occurs in 43.2% of patients with ischaemic stroke [4, 5]. A degree of stenosis of 30–75% may be highly correlated with fatal stroke [5]. Recurrent stroke risks are up to 25–30% 2 years after transient ischaemic attack (TIA) or stroke in patients with a stenosis of 70% or more [6]. Stroke has brought many burdens to countries around the world, particularly low-income countries [7]. Because of the significant social and financial costs associated with intracranial atherosclerosis stenosis, more research is needed to identify and develop optimal treatment strategies.

Primary treatments for symptomatic intracranial stenosis include antithrombotic therapy, endovascular therapy and risk factor control [8]. Risk factor control includes control of hypertension with medications and cholesterol levels with statins. The Warfarin Aspirin Symptomatic Intracranial Disease (WASID) study demonstrated that aspirin was associated with significantly lower rates of death and haemorrhage and provided advantages over warfarin in terms of stroke or death prevention [9]. In patients with symptomatic intracranial atherosclerosis stenosis, short-term dual antiplatelet therapy may be more effective than single aspirin therapy at reducing the high recurrence risk of stroke [10, 11]. Despite antiplatelet agents being the primary therapeutic, their beneficial effect on intracranial atherosclerosis is still modest; as a result, the 1-year risk of ischaemic stroke is still as high as 23% [9, 12]. In the 1980s, endovascular therapy began being considered a potential therapeutic option in patients with symptomatic intracranial atherosclerosis stenosis [13]. It is typically used when patients with > 70% stenosis have ischaemic events despite optimal medical therapy. Endovascular therapy consists of angioplasty and stenting; however, half of the patients treated with angioplasty alone have over 50% postoperative residual stenosis [8, 14]. Combining angioplasty with stenting results in a greater overall improvement in vessel diameter [14]. Thus, for most interventionists, stenting has become the preferred endovascular treatment for patients with symptomatic intracranial stenosis [8]. No RCT comparing patients treated with medical therapy alone to those treated with stenting plus medical therapy was conducted before the Stenting vs. Aggressive Medical Management for Preventing Recurrent Stroke in Intracranial Stenosis (SAMMPRIS) trial in 2013 [15]. The results of the SAMMPRIS trial and the Vitesse Intracranial Stent Study for Ischemic Stroke Therapy (VISSIT) trial supported the use of medical management alone as being superior to stenting plus medical management [15, 16]. There were few indications changed for stenting in cases of intracranial atherosclerosis stenosis [17]. However, studies from other authors indicated promising outcomes for stent therapy. Stenting was found to be safer and more durable, as well as having better functional outcomes, than medical therapy alone in patients with severe symptomatic intracranial stenosis [18].

At present, a total of three RCTs have been published; however, the effect of stenting plus medical therapy versus medical therapy alone for the treatment of patients with intracranial atherosclerosis stenosis remains unclear. Thus, we pooled data from previous RCTs and conducted a meta-analysis to investigate the effect of stenting plus medical therapy versus medical therapy alone in patients with symptomatic intracranial stenosis.

## Methods

### Study protocol

Before the project started, we drafted a research protocol following the Cochrane Collaboration format [19]. The protocol for this systematic review was registered on PROSPERO with the number CRD42022353224.

### Eligibility criteria

We set the inclusion criteria as follows: (a) study type: RCT; (b) language restriction: available only in English; (c) participants: patients ≥ 18 years of age who had symptomatic intracranial stenosis, that is, had nondisabling stroke (modified Rankin Scale score, 0–2) or a TIA that was attributed to severe stenosis (degree of stenosis 70–99%) of a major intracranial artery; (d) intervention: stenting plus medical therapy and medical therapy alone; (e) outcomes: primary outcome including stroke or death within 30 days and within 1 year. Secondary outcomes included any death within 30 days and within 1 year, any ischaemic stroke within 30 days and within 1 year, and any intracranial haemorrhage (ICH) within 30 days and within 1 year. Exploratory outcomes included stroke or death within 2 or 3 years, any death within 2 or 3 years and any ischaemic stroke within 2 or 3 years. The included RCTs were requested to supply all the primary outcomes and at least one secondary efficacy outcome or exploratory outcome mentioned above.

### Search strategy

MEDLINE, EMBASE, the Cochrane Library and ClinicalTrials.gov were systematically searched to identify relevant studies published before August 24, 2022. The following search strategy was employed: (“stenting therapy” OR “endovascular therapy” OR “stenting plus medical therapy” OR “medical therapy”) AND “intracranial artery stenosis” in the title, abstract or keywords. The detailed search strategy can be found in the electronic supplementary material (Table S1). Additionally, the reference lists of RCTs, relevant systematic reviews and meta-analyses were also screened independently and manually to ensure a more comprehensive search.

### Study selection and data collection

According to the eligibility criteria mentioned above, two reviewers (XW and JXL) independently reviewed all titles, abstracts, and full-text articles searched from the four databases and the reference lists of RCTs and relevant systematic reviews or meta-analyses. We excluded duplicates and research articles for which the full text was not available. Discrepancies between the two authors were resolved by discussion or, if necessary, by a third author (SXW) who did not participate in the data collection. After selection and evaluation, all data from the included RCTs were extracted as follows: basic information and outcome events included for each RCT (Table 1); inclusion and exclusion criteria, the study design, and all efficacy and safety outcomes are shown in the online supplementary materials (Table S2).

**Table 1:** Characteristics of the included randomized controlled trials for patients with symptomatic intracranial stenosis and outcome events

Study	Countries	Centers	Outcome events	Treatment group (no. of participants)	Male (%)	Mean age ± SD (years)	Mean time ± SD from latest ischemic event to randomization (days)	Qualifying event	Arterial stenosis (mean ± SD, %)
Derdeyn et al. [15] (NCT00576693) (SAMMPRIS)	USA	50	a, e, f, g, h, i, j, k	SPMT (224)	56.7	61.0 ± 10.7	9.1 ± 9.0	TIA 82	80.0 ± 7.0
								Stroke 142	
				MT (227)	63.9	59.5 ± 11.8	10.2 ± 11.2	TIA 75	81.0 ± 7.0
								Stroke 152	
Zaidat et al. [16] (NCT00816166) (VISSIT)	USA	27	a, b, c, d, e, f, g	SPMT (58)	70.7	61.8 ± 12.3	12.3 ± 9.6	TIA 24	78.9 ± 7.3
								Stroke 36	
				MT (53)	60.4	61.8 ± 12.8	15.2 ± 10.3	TIA 22	80.4 ± 7.5
								Stroke 34	
Gao et al. [22] (NCT01763320) (CASSISS)	China	8	a, b, c, d, e, f, g, h, i, j, k	SPMT (176)	72.7	56.7 ± 9.4	42.8 ± 28.8	TIA 87	78.4 ± 6.4
								Stroke 89	
				MT (182)	74.2	55.9 ± 9.8	44.4 ± 29.9	TIA 77	76.9 ± 5.8

*SPMT* stenting plus medical therapy, *MT* medical therapy, *TIA* transient ischemic attack; a: any stroke or death within 30 days; b: any death within 30 days; c: any ischemic stroke within 30 days; d: intracranial hemorrhage within 30 days; e: any stroke or death within 1 year; f: any death within 1 year; g: any ischemic stroke within 1 year; h: intracranial hemorrhage within 1 year; i: stroke or death within 2 or 3 years; j: any death within 2 or 3 years; k: any ischemic stroke within 2 or 3 years

### Risk of bias

The risk of bias plot was evaluated with Review Manager 5.3 software. The uniform criteria of the Cochrane Collaboration were used to assess the risk of bias for RCTs [20], which included selection bias, performance bias, detection bias, attrition bias, reporting bias, and other potential biases. Each bias criterion was classified as “low”, “high”, or “unclear”. The assessment was carried out independently by XW and JXL. Disagreements were settled by consulting with a third author (SXW).

### Summary measures and synthesis of results

We used Review Manager 5.3 software to perform pairwise meta-analysis of direct evidence. The relative risk (RR) with 95% confidence interval (95% CI) was analysed and calculated for the dichotomous outcomes. We then estimated the heterogeneity through the *I*^2^ statistic as follows: *I*^2^ < 30% suggests “low heterogeneity”; *I*^2^ between 30 and 50% indicates “moderate heterogeneity”; *I*^2^ > 50% denotes “substantial heterogeneity”. The data were analysed with a fixed effects model for those with less than 50% heterogeneity, and for those with greater than 50% heterogeneity, we used the random effects model. A sensitivity analysis was also carried out to explore the stability of the consolidated results. We also implemented subgroup analyses to detect the effect of different times from the qualifying event to randomization (> 3 weeks and < 3 weeks) and different types of stents (wingspan stent and balloon-expandable stent [BES]) at baseline on primary outcomes. For all the analyses, two-tailed tests were performed, and a *P* value < 0.05 was considered to be statistically significant.

## Results

**Fig. 1:**
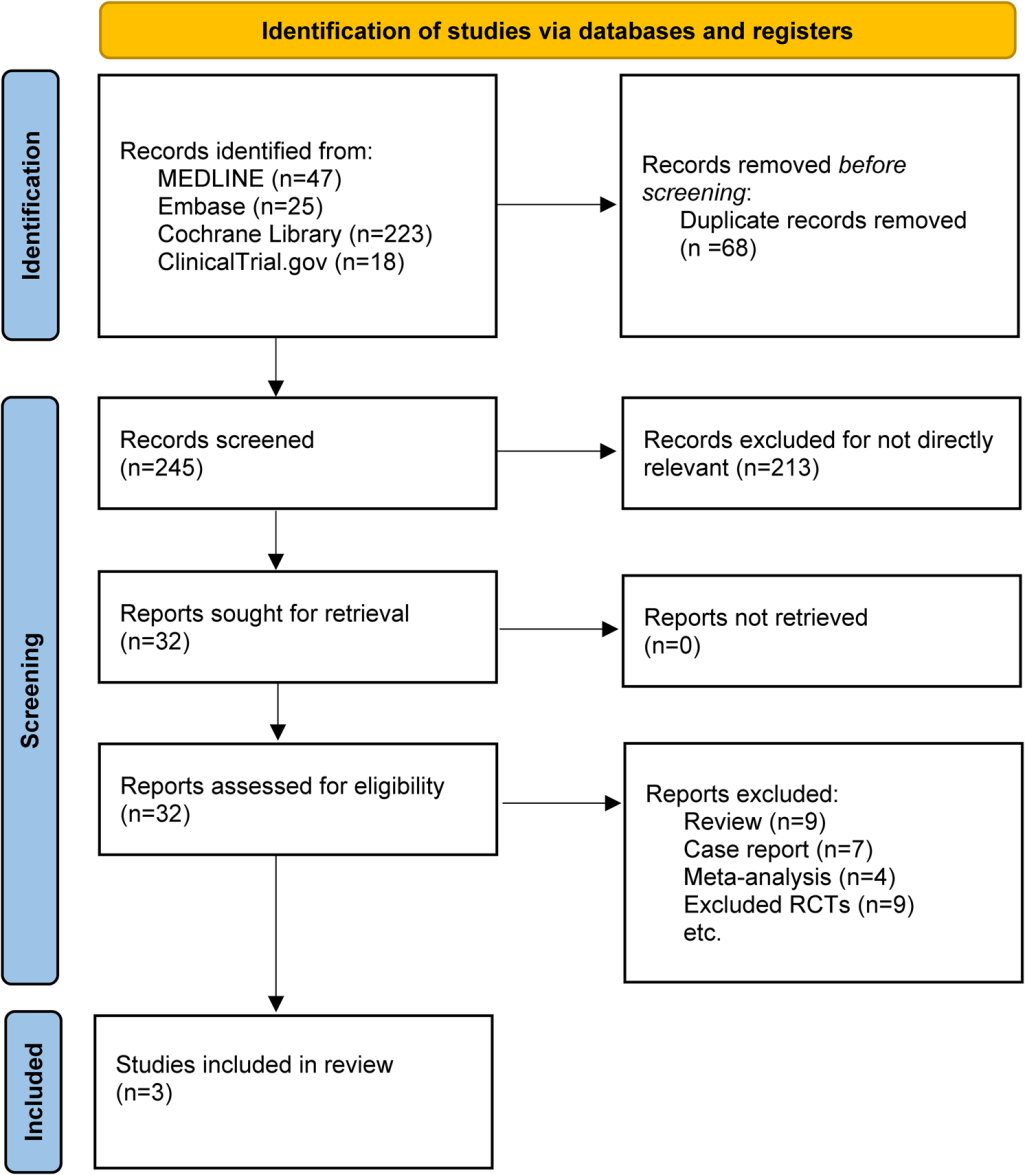
The study search, selection, and inclusion process

MEDLINE, EMBASE, the Cochrane Library, and Clinicaltrials.gov together provided 313 titles and abstracts. A total of 281 articles were excluded due to duplication and irrelevance after a quick review, and 32 full articles were assessed for eligibility. Among these, 29 articles were excluded due to the inapplicable publication types: Nine were non-RCTs, seven were case reports, four were meta-analyses and nine were reviews. The selection process is summarized in the flow diagram (Fig. 1). The main characteristics of the three included studies are summarized in Table 1.

### Primary outcome analysis

**Fig. 2:**
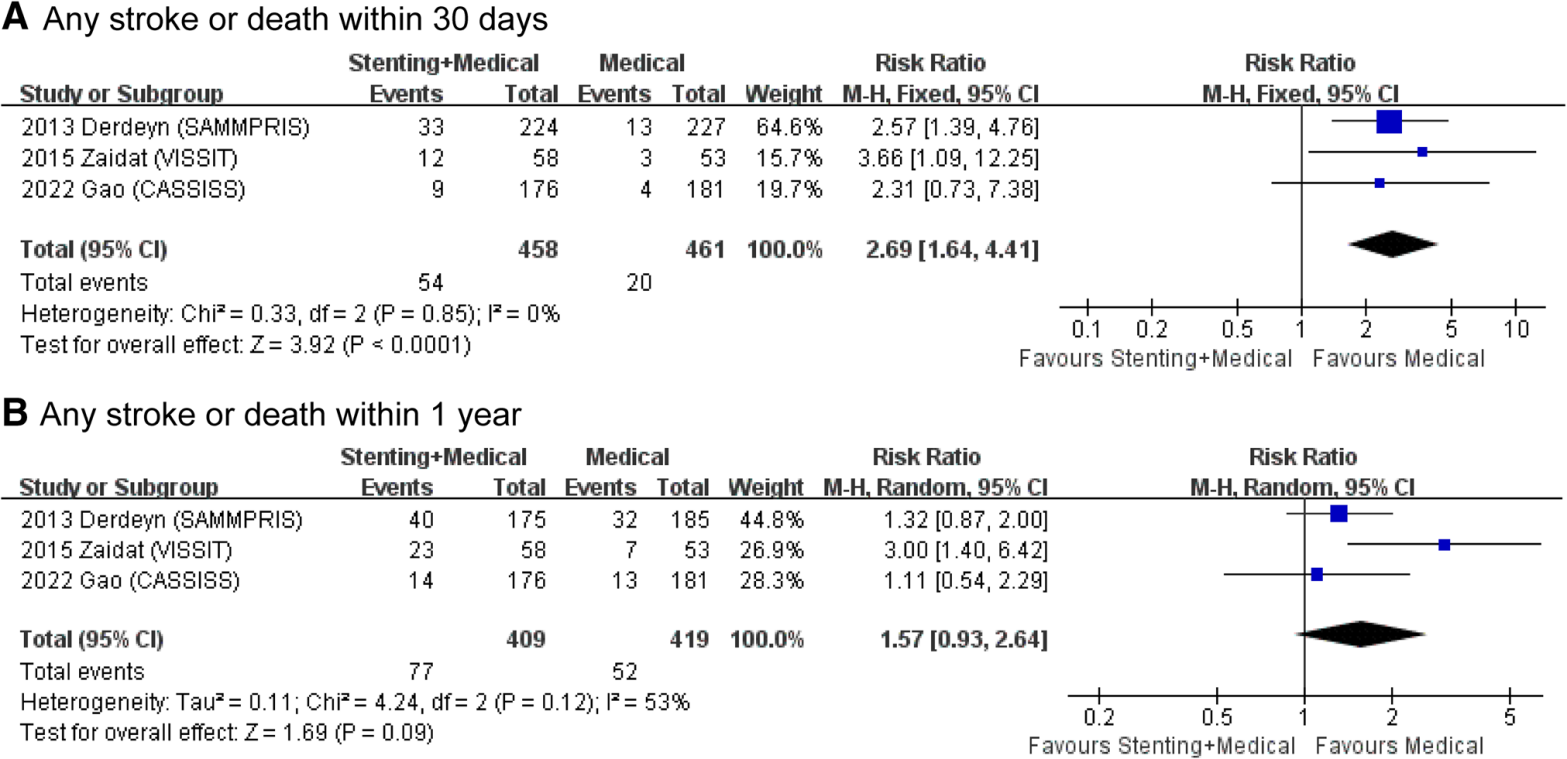
Forest plots for primary outcomes

As shown in Fig. 2A, patients in the stenting plus medical therapy group had a significantly higher risk of stroke or death within 30 days than those in the medical therapy only group (RR = 2.69 [1.64–4.41], *P* < 0.0001, *I*^2^ = 0%). However, when the follow-up time was extended to 1 year, there was no statistically significant difference in the risk of any stroke or death among patients receiving additional stent implantation compared with those receiving medical therapy alone (RR = 1.57 [0.93–2.64], *P* = 0.09, Fig. 2B). High heterogeneities were found in this outcome (*I*^2^ = 53%); therefore, sensitivity analyses were performed, and they demonstrated that all the statistics were robust (Fig. S1).

### Secondary and exploratory outcome analysis

**Fig. 3:**
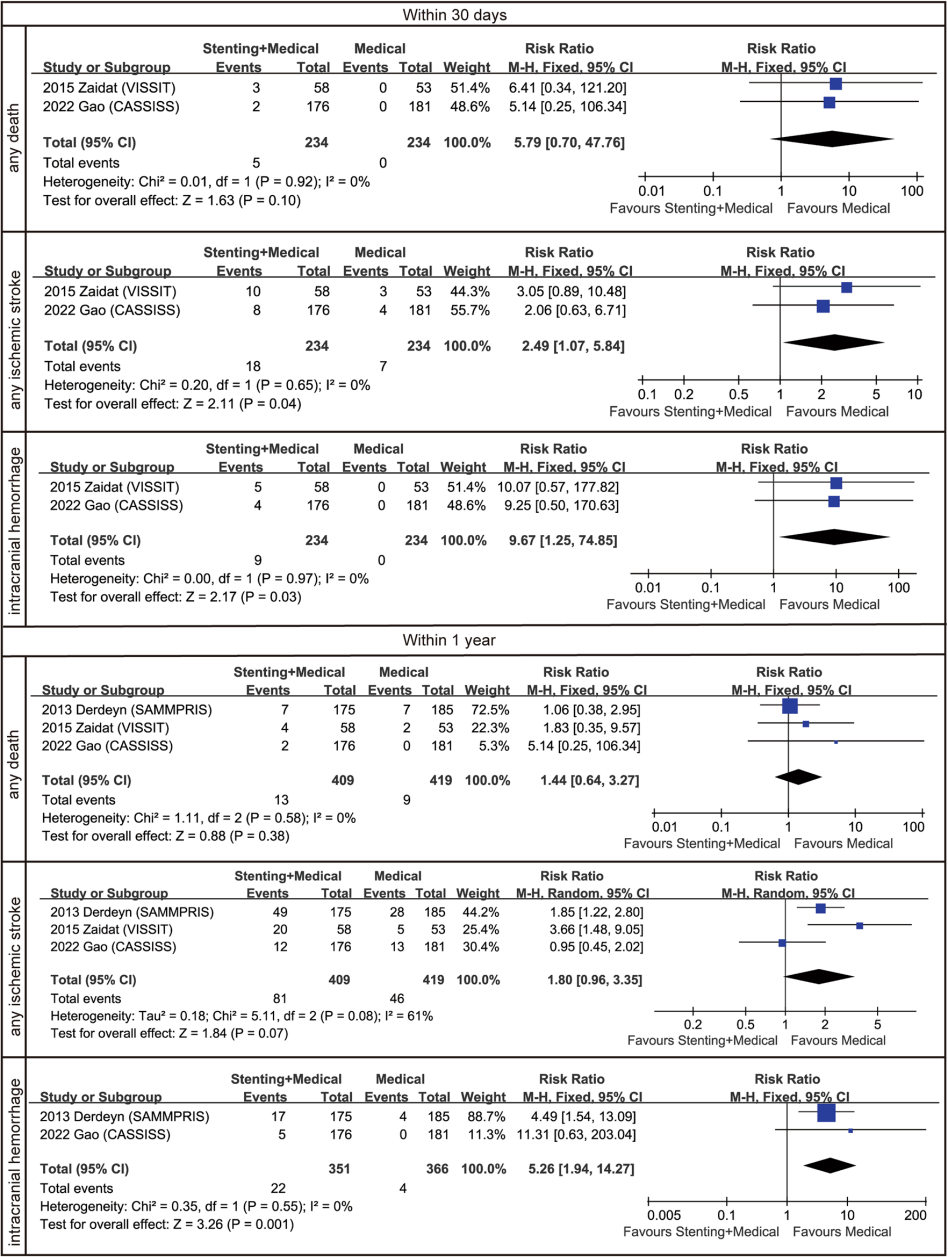
Forest plots for secondary outcomes

For secondary outcomes within 30 days, we were not able to collect data on the SAMMPRIS trial. As shown in Fig. 3, no significant differences were found between the stenting plus medical therapy group and the medical therapy only group regarding the mortality rate either within 30 days or within 1 year (*P* = 0.10 and 0.38, respectively). In contrast, compared with medical therapy only, stenting plus medical therapy put patients at a higher risk of intracranial haemorrhage (within 30 days: RR = 9.67 [1.25, 74.85], *P* = 0.03; within 1 year: RR = 5.26 [1.94, 14.27], *P* = 0.001). For any ischaemic stroke, the high risk in the stenting plus medical therapy group was found only within 30 days (RR = 2.49 [1.07, 5.84], *P* = 0.04). Figure 3 shows the detailed results of the secondary outcome analysis. Sensitivity analysis was also performed for any ischaemic stroke within 1 year (*I*^2^ = 61%), and it demonstrated that if we excluded the CASSISS trial, the overall heterogeneity would have been less than 50%; at the same time, the medical therapy alone group would show a lower risk of ischaemic stroke (Fig. S2).

**Fig. 4:**
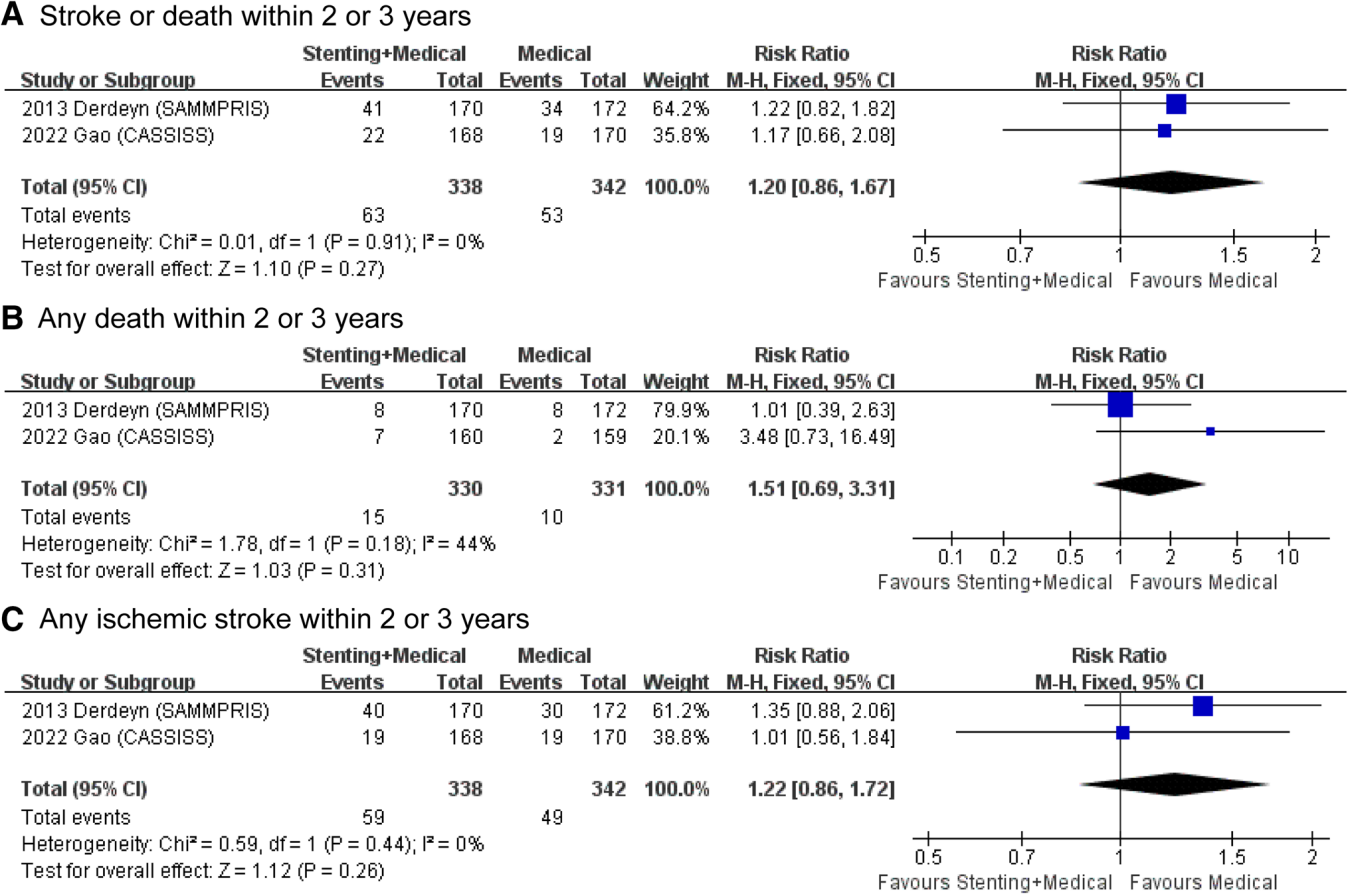
Forest plots for exploratory outcomes

Exploratory outcomes included stroke or death within 2 or 3 years, any death within 2 or 3 years and any ischaemic stroke within 2 or 3 years. Since the VISSIT trial had a maximum follow-up of only 1 year, only data from the SAMMPRIS and CASSISS trials were analysed for exploratory outcomes. The results showed that there were no significant differences between the stenting plus medical therapy group and the medical therapy only group for all exploratory outcomes (Fig. 4).

### Subgroup analyses

To assess the influence of different times from the qualifying event to randomization (> 3 weeks and < 3 weeks) and different types of stents (wingspan stent and BES), we implemented subgroup analyses at baseline. The results indicated that, if the time from the qualifying event to randomization was extended to > 3 weeks, additional stenting would have no effect on any stroke or death, whether within 30 days or within 1 year (*P* = 0.16 and 0.78, respectively). In the subgroup analysis of different types of stents, we found that the risk of any stroke or death within 1 year was still significantly higher for those patients implanted with a BES than those patients in the medical therapy only group (RR = 3.00 [1.40, 6.42], *P* = 0.005). The detailed results of the subgroup analyses are shown in Table 2 and Figs. S3–S6.

**Table 2:** Subgroup analysis of primary outcomes

	Stroke or death within 30 days		Stroke or death within 1 year	
	RR [95% CI]	*p* value	RR [95% CI]	*p* value
*Time from latest ischemic event to randomization*				
> 3 weeks	2.31 [0.73, 7.38]	0.16	1.11 [0.54, 2.29]	0.78
< 3 weeks	2.78 [1.61, 4.81]	0.0002	1.87 [0.84, 4.15]	0.12
*Stent type*				
Wingspan stent	2.51 [1.46, 4.32]	0.0009	1.26 [0.88, 1.81]	0.21
Balloon-expandable stent	3.66 [1.09, 12.25]	0.04	3.00 [1.40, 6.42]	0.005

*RR* Relative Risk, *CI* confidence interval

### Risk of bias in included studies

**Fig. 5:**
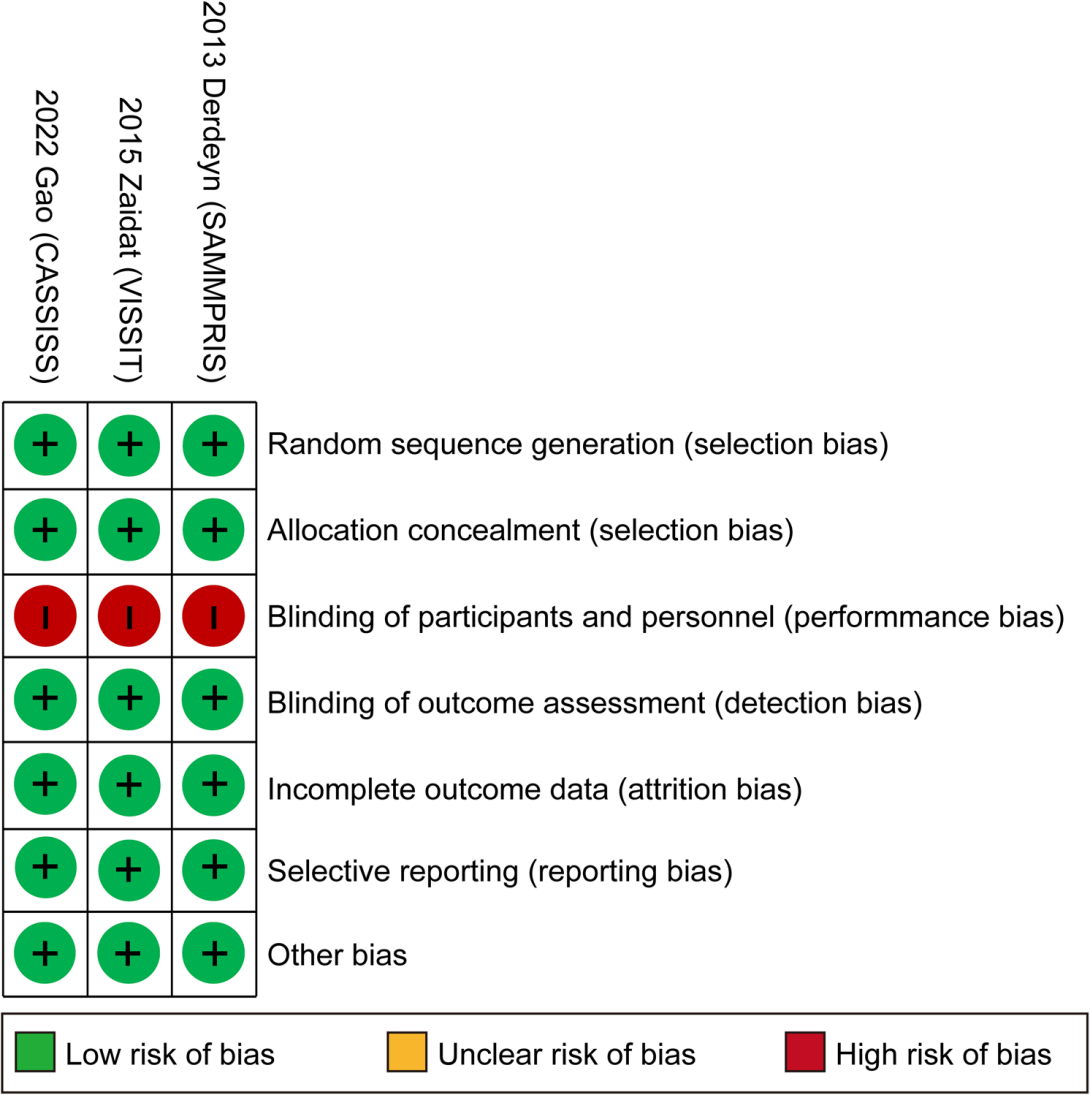
Risk of bias: a summary table for each risk of bias item for each study

The risk of bias for all enrolled studies is illustrated in Fig. 5. All three included RCTs showed a low risk of bias in random sequence generation and allocation concealment. The risk for performance bias was considered high in all RCTs, as blinding of the participants and personnel was not possible. For blinding of outcome assessment, the risk of bias was low in all trials. For incomplete outcome data and selective reporting, the risk of bias was also low in all three studies. Aside from these items, no other risk of bias was observed.

## Discussion

Stroke is a major contributor to mortality globally, and intracranial atherosclerosis stenosis is the main cause of stroke [4, 21]. Stenting and medical management are used to prevent recurrent ischaemic stroke in patients with symptomatic severe intracranial atherosclerotic stenosis. Nevertheless, there is no agreement on the optimal treatment strategy for patients with intracranial atherosclerosis stenosis. According to the American Heart Association Guidelines, medical management with antiplatelet therapy and risk factor control is recommended for patients with stroke or TIA due to 50–99% intracranial stenosis [12], as the value of stent implantation is uncertain. Recently, an RCT reported by Gao et al. systematically compared stenting plus medical therapy with medical therapy alone [22]. Consistent with their findings, our meta-analysis results showed that the effect of stenting plus medical therapy on the risk of stroke and death was not inferior to that of medical therapy alone in the long term.

This systematic review and meta-analysis included three RCTs with 921 participants presenting symptomatic intracranial atherosclerotic stenosis with a degree of stenosis ≥ 70%. Our present study demonstrated that, in terms of 30-day death or stroke, ischaemic stroke, intracranial haemorrhage, and one-year intracranial haemorrhage, medical therapy alone was significantly superior to stenting plus medical therapy. However, there were no significant differences in terms of 1-year and up to 3-year outcomes in terms of death or stroke, death, and ischaemic stroke. These results show that the benefit of medical therapy over stenting plus medical therapy for high-risk patients with intracranial atherosclerotic stenosis no longer exists in the long term. This may be due to the perioperative complications of stent implantation and the increased risk of intracerebral haemorrhage caused by stenting plus dual antiplatelet therapy [23]. Interestingly, we observed high heterogeneity (*I*^2^ = 61%) in the outcome of any ischaemic stroke within 1 year; therefore, to better evaluate heterogeneity, we performed a sensitivity analysis by removing individual studies. It demonstrated that the overall heterogeneity would have been less than 50% without the China Angioplasty and Stenting for Symptomatic Intracranial Severe Stenosis (CASSISS) trial; meanwhile, the medical therapy alone group would show a lower risk of ischaemic stroke. Patients enrolled in CASSISS had a time interval from the qualifying event to stenting of more than 3 weeks, which was significantly longer than that for SAMMPRIS and VISSIT. There were differences in the ethnicity of participants between CASSISS versus SAMMPRIS and VISSIT. These factors may be associated with lower perioperative complications and risk of ischaemic stroke [24–26].

Further subgroup analyses showed that the time interval from the qualifying event to intervention as well as the type of stents may be factors affecting the outcomes of stent treatment. According to the subgroup analysis, stent implantation performed more than 3 weeks after the qualifying event appears to be safer than when performed less than 3 weeks after the qualifying event. However, the detailed analysis of SAMMPRIS results demonstrated that there is no correlation between the rate of outcomes and the amount of time between the qualifying event and the intervention [27]. Our result was consistent with previous studies, which indicated that stenting early after the ischaemic event carries considerable risk [25, 26, 28–30]. The cause of increased risk may be attributed to plaque instability and reperfusion injury [31]. Early operation on recently symptomatic vulnerable plaques is more likely to result in plaque movement and embolism than delayed interventions on stabilized plaques [30]. Instability of the intracranial microcirculation and impairment of cerebral autoregulation in the area of ischaemic stroke may lead to an increased risk of postoperative haemorrhage after surgery in patients with symptomatic severe intracranial atherosclerotic stenosis treated in the acute or subacute phase [32]. When the perfusion pressure suddenly increases, the arterioles are vulnerable to rupture and bleeding [32]. Thus, the time interval from the ischaemic event to intervention should be taken into account in future studies on the safety and efficacy of stenting for symptomatic intracranial atherosclerotic stenosis.

Another subgroup analysis revealed that for patients with severe intracranial stenosis, the risk of any stroke or death within 1 year was still significantly higher for those patients implanted with a BES than those patients in the medical therapy alone group. A meta-analysis comparing the outcomes of various types of endovascular therapy was published in 2021. Wang et al. concluded that, compared to medical therapy, both types of stents were associated with a high risk of 30-day death or stroke, with no significant intergroup difference [25]. However, they did not analyse the outcomes of various types of stenting during the long-term follow-up. The Wingspan stent is a self-expandable stent that has been the most widely used and approved by the Food and Drug Administration (FDA) [31, 33]. The Vitesse is a BES that is limited as an investigational device in the US [33]. However, previous studies have reported that intracranial stenting with a BES is more effective in reducing the degree of stenosis and has a lower complication rate than a Wingspan stent [34–36]. This rather contradictory result may be due to several potential explanations. Wingspan stents are more flexible than BESs and thus would be suitable in tortuous vessels. In contrast, a BES has higher rigidity and is thus easier to deploy [29, 37]. The major symptomatic qualifying arteries of patients in the included two RCTs were the middle cerebral artery and the basilar artery, which are prone to perforator occlusion during balloon expansion due to the tortuous path and numerous perforators [15, 22]. The VISSIT did not report the proportion of different qualifying arteries [16]. Our analysis was based on limited RCTs, and only one study used BESs; thus, the results are not definitive. More clinical studies are needed in the future to explore the choice of types of stenting.

The results of our study may have been influenced by several limitations. First, due to the small number of RCTs included, there is a lack of comprehensive persuasiveness. Only three published RCTs were pooled despite a thorough search to compare the effectiveness of stenting plus medical therapy versus medical therapy alone. Second, heterogeneity existed between the RCTs with respect to the inclusion criteria and exclusion criteria for participants and operators, baseline characteristics (e.g., mean age, sex, and ethnicity), the dose of aspirin and the time between the qualifying event and randomization, which may have contributed to our statistics being discrepant. Two trials were stopped earlier than planned because of safety concerns regarding the risk of stroke or death in the stenting plus medical therapy group. Third, a previous study indicated that sex differences could lead to different outcomes of stroke. Arboix et al. found that compared with men, the mean age at stroke occurrence in women was older, and women presented more frequently with hypertension and atrial fibrillation. Cardioembolic stroke was more common in women, while men were more likely to have a lacunar stroke. In addition, women had worse early outcomes, higher in-hospital mortality, and longer hospital stays and were more disabled than men [38]. However, we could not perform a subgroup analysis based on sex since the original individual data were not accessible. Therefore, this meta-analysis cannot provide clinicians evidence regarding the differences in the risk of stroke and death in both groups with respect to sex. In addition, all the RCTs included were considered to have a high risk of performance bias. Finally, long-term comparisons of the effect of stenting plus medical therapy versus medical therapy alone are still lacking, and the included RCTs had a maximum follow-up of only 36 months.

## Conclusion

In conclusion, the present study indicated that for patients with symptomatic intracranial stenosis, medical therapy alone has a lower risk of stroke and death in the short term than stenting plus medical therapy, while no difference exists in long-term follow-up between 1 and 3 years. At the same time, the timing of stent implantation and the type of stent may also affect the risk of stroke and death for additional stenting operations. More studies, especially those with long-term follow-ups, are needed to identify precision strategies for patients with symptomatic intracranial stenosis.

## Supplementary Information

Below is the link to the electronic supplementary material.

Supplementary file1 (DOCX 944 KB)

## Data Availability

All data generated or analyzed during this study are included in this published article and its supplementary information files.
