# Supplementary material for: Comparative effect of stenting plus medical therapy vs medical therapy alone on the risk of stroke and death in patients with symptomatic intracranial stenosis: a systematic review and meta-analysis
Source: J Neurol. 2022 Oct 27;270(2):662–72. doi: 10.1007/s00415-022-11429-9 (PMC9886616; doi:10.1007/s00415-022-11429-9)
Supplement: Supplementary file 1 — Supplementary file1 (DOCX 944 KB) [file 415_2022_11429_MOESM1_ESM.docx]

Journal name: Journal of Neurology

Comparative effect of stenting plus medical therapy vs medical therapy alone on the risk of stroke and death in patients with symptomatic intracranial stenosis: a systematic review and network meta-analysis

Xin Wu ^1,2 #^, Jiaxuan Li ^2, #^, Shixin Wang ^2, #^, Yu Zou ^1^, Liyan Tang ^2^, Zhouqing Chen ^2^, Wei Zhang ^1, *^, Zhong Wang ^2, *^

^1^ Department of Neurosurgery, Suzhou Ninth People’s Hospital, Suzhou, Jiangsu Province, China

^2^ Department of Neurosurgery & Brain and Nerve Research Laboratory, The First Affiliated Hospital of Soochow University, Suzhou, Jiangsu Province, China

^#^ Xin Wu, Jiaxuan Li and Shixin Wang contribute equally to this work.

*** Correspondence:**Zhong Wang, Department of Neurosurgery, The First Affiliated Hospital of Soochow University, 188 Shizi Street, Suzhou 215006, China. Email address: wangzhong761@163.com.

Wei Zhang, Department of Neurosurgery, Suzhou Ninth People’s Hospital, Suzhou, 215200, China. Email address: mrzhangweish@163.com.

**Figure legends**

Figure S1: Sensitivity analysis of the data with heterogeneity greater than 50%: any stroke or death within 1 year. **A.** Any stroke or death within 1 year without SAMMPRIS trial. **B.** Any stroke or death within 1 year without VISSIT trial. **C.** Any stroke or death within 1 year without CASSISS trial.

Figure S2: Sensitivity analysis of the data with heterogeneity greater than 50%: any ischemic stroke within 1 year. **A.** Any ischemic stroke within 1 year without SAMMPRIS trial. **B.** Any ischemic stroke within 1 year without VISSIT trial. **C.** Any ischemic stroke within 1 year without CASSISS trial.

Figure S3: Sub-analysis of time from qualifying event to randomization in primary outcome: stroke and death within 30 days.

Figure S4: Sub-analysis of time from qualifying event to randomization in primary outcome: stroke and death within 1 year.

Figure S5: Sub-analysis of stent type in primary outcome: stroke and death within 30 days.

Figure S6: Sub-analysis of stent type in primary outcome: stroke and death within 1 year.

Table S1: The detailed search strategy.

Table S2: Inclusion, exclusion criteria, study design and outcome assessments of the included studies.

**Table S1: Detailed Search Strategy**

Search Date: 24 Aug 2022

**Pubmed：**

| Search | Query | Results |
| --- | --- | --- |
| #1 | "symptomatic intracranial stenosis"[All Fields] | 1,789 |
| #2 | "Drug Therapy"[Mesh] | 1,472,258 |
| #3 | (((((((Therapy, Drug[Title/Abstract]) OR (Drug Therapies[Title/Abstract])) OR (Therapies, Drug[Title/Abstract])) OR (Chemotherapy[Title/Abstract])) OR (Chemotherapies[Title/Abstract])) OR (Pharmacotherapy[Title/Abstract])) OR (Pharmacotherapies[Title/Abstract])) OR (medical therapy[Title/Abstract]) | 498,790 |
| #4 | #2 OR #3 | 1,812,286 |
| #5 | "Stents"[Mesh] | 87,099 |
| #6 | (Stent[Title/Abstract]) OR (stenting[Title/Abstract]) | 102,651 |
| #7 | #5 OR #6 | 124,743 |
| #8 | #4 OR #7 | 1,926,872 |
| #9 | #1 AND #8 | 913 |
| #10 | "Randomized controlled trial"[Filter] | 576181 |
| #11 | #9 AND #10 | **47** |

**Embass：**

| Search | Query | Results |
| --- | --- | --- |
| #1 | 'drug therapy'/exp | 3,305,311 |
| #2 | 'medical therapy':ab,ti,kw | 50,112 |
| #3 | 'therapy, drug':ab,ti,kw | 1,444 |
| #4 | 'drug therapies':ab,ti,kw | 7,325 |
| #5 | 'therapies, drug':ab,ti,kw | 146 |
| #6 | 'chemotherapy':ab,ti,kw | 686,528 |
| #7 | 'chemotherapies':ab,ti,kw | 12,363 |
| #8 | 'pharmacotherapy':ab,ti,kw | 55,488 |
| #9 | 'pharmacotherapies':ab,ti,kw | 5,784 |
| #10 | #1 OR #2 OR #3 OR #4 OR #5 OR #6 OR #7 OR #8 OR #9 | 3,582,823 |
| #11 | 'stent'/exp | 203,208 |
| #12 | 'stents':ab,kw,ti | 71,920 |
| #13 | 'stenting':ab,kw,ti | 61,753 |
| #14 | #11 OR #12 OR #13 | 222,159 |
| #15 | 'symptomatic intracranial stenosis' | 267 |
| #16 | #10 OR #14 | 3,769,457 |
| #17 | #15 AND #16 | 194 |
| #18 | 'random':ab,ti OR 'control':ab,ti OR 'double-blind':ab,ti | 4,342,980 |
| #19 | #17 AND #18 | **25** |

**Cochrane:**

| Search | Query | Results |
| --- | --- | --- |
| #1 | MeSH descriptor: [Drug Therapy] explode all trees | 148242 |
| #2 | (medical therapy):ti,ab,kw OR (Therapy, Drug):ti,ab,kw OR (Drug Therapies):ti,ab,kw OR (Therapies, Drug):ti,ab,kw OR (Chemotherapy):ti,ab,kw | 564218 |
| #3 | (Chemotherapies):ti,ab,kw OR (Pharmacotherapy):ti,ab,kw OR (Pharmacotherapies):ti,ab,kw | 91828 |
| #4 | #1 OR #2 OR #3 | 604334 |
| #5 | MeSH descriptor: [Stents] explode all trees | 4567 |
| #6 | (Stent):ti,ab,kw OR (stenting):ti,ab,kw | 16985 |
| #7 | #5 OR #6 | 16985 |
| #8 | #4 OR #7 | 614607 |
| #9 | (symptomatic intracranial stenosis) | 327 |
| #10 | #8 AND #9 | **223** |

**Clinicaltrials.gov：**

| Search | Query | Results |
| --- | --- | --- |
| #1 | Status:All studies,condition or disease: Symptomatic intracranial stenosis, and other terms: Medical therapy, Study type: interventional(Clinical Trial) | 12 |
| #2 | Status:All studies,condition or disease: Symptomatic intracranial stenosis,and other terms: Stents, Study type: interventional(Clinical Trial) | 6 |
| Total |  | **18** |

**Table S2: Inclusion, exclusion criteria, study design, and outcome assessments of the included studies**

| **Trials** | **Derdeyn et al 2013 (SAMMPRIS, NCT00576693)** |
| --- | --- |
| ***Inclusion Criteria*** | Eligible patients were between 30 and 80 years with TIA or non-severe stroke within 30 days of enrollment attributed to 70-99% stenosis of a major intracranial artery (carotid artery, MCA stem (M1), vertebral artery, or basilar artery) must be confirmed by catheter angiography; mRS of ≤ 3; Target area of stenosis in an intracranial artery that has a normal diameter of 2.00 mm to 4.50 mm; Target area of stenosis is less than or equal to 14 mm in length; Female patients had normal menses in the last 18 months and no pregnancy; Patient can return for all follow-up visits and understands the purpose and requirements of the study; |
| ***Exclusion Criteria*** | Tandem extracranial or intracranial stenosis (70%-99%) or occlusion that is proximal or distal to the target intracranial lesion; Bilateral intracranial vertebral artery stenosis of 70%-99% and uncertainty about which artery is symptomatic; Endovascular therapy of an extracranial (carotid or vertebral artery) or intracranial artery within 30 days before enrollment or of target lesion previously; Plan to perform concomitant angioplasty or stenting of an extracranial vessel tandem to intracranial stenosis; Presence of intraluminal thrombus or aneurysm or calcification proximal to or at the target lesion; Intracranial tumor (except meningioma) or any intracranial vascular malformation; Thrombolytic therapy or progressive neurological signs within 24 hours prior to enrollment; Brain infarct size > 5 cm within 30 days prior to enrollment; Any hemorrhagic infarct within 14 days or intracranial hemorrhage within 30 days; Intracranial arterial stenosis due to arterial dissection, moya moya disease; any known vasculitis disease; herpes zoster, varicella zoster or other viral vasculopathy; neurosyphilis; any other intracranial infection; any intracranial stenosis associated with CSF pleocytosis; radiation induced vasculopathy; fibromuscular dysplasia; sickle cell disease; neurofibromatosis; benign angiopathy of central nervous system; post-partum angiopathy; suspected vasospastic process, suspected recanalized embolus; Presence of any unequivocal cardiac sources of embolism; Known allergy or contraindication to aspirin, clopidogrel, heparin, nitinol, contrast dye, local or general anesthesia; Active bleeding diathesis; Undergone or had have major surgery (including open femoral, aortic, or carotid surgery); Indication for warfarin or heparin beyond enrollment; Severe condition that would prevent participation in study procedures or with life expectancy within 3 months; Pregnancy or unwilling to use contraception for the duration of this study; |
| ***Study Design*** | A Multicenter, prospective, randomized, controlled, open-label clinical trial, comparing aggressive medical management alone or aggressive medical management plus PTAS with the use of the Wingspan stent system |
| ***Primary Outcomes*** | Any stroke or death within 30 days; Ischemic stroke in the territory of the qualifying artery beyond 30 days |
| ***Secondary And Safety Outcomes*** | Any stroke or death; Myocardial infarction; Disabling or fatal stroke; Major non-stroke hemorrhage; Any major hemorrhage |

| **Trials** | **Zaidat et al 2015 (VISSIT, NCT00816166)** |
| --- | --- |
| ***Inclusion Criteria*** | Subject between 18 and 85 years has at least one neurovascular lesion (70-99%) stenosis [internal carotid, middle cerebral, vertebral artery (C4-BA), and/or basilar artery] symptomatic with a hard TIA or stroke attributable to the territory of the lesion within the past 30 days; An intracranial tandem lesion (50-99%) stenosis may be treated if normal artery segment is sufficient length to avoid overlapping stents; Target vessel diameter / lesion length measurements are within one of the below per angiogram: vessel diameter is >=2.0 mm and < 2.5 mm / lesion length is <= 16 mm, or vessel diameter is >= 2.5 mm and < 3.0 mm / lesion length is <= 18 mm, or vessel diameter is >= 3.0 mm and < 4.5 mm / lesion length is <= 26 mm, or vessel diameter is >= 4.5 mm and <= 5.0 mm / lesion length is <= 36 mm; Subject has normal artery adjacent to each stenosis, diameter 2.0 mm - 5.0 mm; Life expectancy is at least 2 years; mRS score is <= 3; Subject is available for study follow-up visits and provide Informed Consent |
| ***Exclusion Criteria*** | Subject has contraindications for balloon expandable stent, e.g. a. Extreme tortuosity at, or proximal to, target lesion, b. More than 2 lesions with > 50% stenosis (including vertebral ostia and common carotid disease), c. Carotid or vertebral dissection; CT scan or MRI evidence of any of the following: a. Intracranial hemorrhage of type PH1 or PH21 b. Subdural or epidural hemorrhage c. Mass effect, or d. Intracranial tumor (except small meningioma); Subject has a previous stent in the territory of the target lesion(s); Subject has a previous coil or clip placed in the territory of the target lesion within 6 months; Subject has a potential source of cardiac embolism requiring anticoagulation therapy; Subject has concurrent intracranial pathology, e.g. a. moya moya b. Vasculitis documented by biopsy results c. Ruptured Aneurysm d. Unruptured aneurysm > 7mm; Subject has uncontrolled hypertension (systolic >185 mmHg or diastolic >110 mmHg); Hemoglobin < 10 g/dL; platelet count < 100,000; INR > 1.5; Subject has an uncorrectable bleeding diathesis; Subject’s NIHSS score increased > 4 points within 48 hours prior to randomization; Subject has a contraindication for combination antithrombotic treatment (e.g., clopidogrel and aspirin); Subject is pregnant or plans to become pregnant in the next 12 months; Myocardial infarction within past 3 months; Treatment with tPA or other thrombolytic agent within 48 hours prior to randomization; Major surgery or trauma within 2 weeks prior to randomization; Enrollment in another investigational device or drug study that may confound the results |
| ***Study Design*** | An international, multicenter, 1:1 randomized, parallel-group trial that enrolled patients from 27 sites, to evaluate the efficacy and safety of the balloon-expandable stent plus medical therapy vs medical therapy alone in patients with symptomatic intracranial stenosis (≥70%) |
| ***Primary Outcomes*** | Stroke in the same territory (distal to the target lesion) as the presenting event within 12 months of randomization; Hard TIA in the same territory (distal to the target lesion) as the presenting event from day 2 through month 12 post randomization |
| ***Secondary And Safety Outcomes*** | Disabling stroke at 1 year (mRS>3); Stent Success – PHAROS Vitesse stent deployed across target lesion with residual stenosis 0-20%; Percentage of Stent Group Subjects with restenosis 50% and 70% confirmed by angiogram at 12 months; NIHSS scores; mRS scores; Any stroke within 30 days of randomization; Hard TIA from day 2 through day 30; Intracranial hemorrhage within 30 days of randomization; Death from any cause within 30 days of randomization; |

| **Trials** | **Gao et al 2022 (NCT01763320)** |
| --- | --- |
| ***Inclusion*** ***Criteria*** | Eligible patients were between 30 and 80 years with transient ischemic stroke (TIA) or stroke within the past 12 months attributed to 70%-99% stenosis of a major intracranial artery (ICA, MCA [M1], vertebral artery, or basilar artery [BA]), stenosis degree must be confirmed by catheter angiography;  MRI scan excluded perforator occlusion; At least 3 weeks intervals from the last symptom of ischemia; No recent infarctions identified on MRI upon enrollment; No massive cerebral infarction (>1/2 MCA territory), intracranial hemorrhage, epidural or subdural hemorrhage, and intracranial brain tumor on CT or MRI scan; mRS <=2; Target vessel reference diameter must be measured to be 2.00 mm to 4.50 mm; target area of stenosis is <=14 mm in length; Female patients had normal menses in the last 18 months and no pregnancy; Patient can return for all follow-up visits and understand the purpose and requirements of the study |
| ***Exclusion Criteria*** | Patients with intracranial arterial stenosis related to the following non-atherosclerotic factors; Impede angiographic assessment; Tandem extracranial or intracranial stenosis (70%–99%) or occlusion that is proximal or distal to the target intracranial lesion; Bilateral intracranial VA stenosis of 70%–99% and uncertainty about which lesion is symptomatic; Plan to perform concomitant angioplasty or stenting of an extracranial vessel tandem to an ipsilateral intracranial stenosis; Endovascular therapy of an extracranial (carotid or vertebral artery) or intracranial artery within 30 days before enrollment or of target lesion previously; Presence of intraluminal thrombus or aneurysm or calcification proximal to or at the target lesion; Intracranial tumors or any intracranial vascular malformations; Thrombolytic therapy or progressive neurological signs within 24 hours prior to enrollment; Brain infarct size > 5 cm within the past 15 days; Previous intracranial hemorrhage within 30 days; Myocardial infarction within previous 30 days; Atrial fibrillation; Intolerance or allergic reaction to any of the medical therapy, including aspirin, clopidogrel, heparin, and local or general anesthetics; Active bleeding diathesis or coagulopathy; Major surgery (including open femoral, aortic, or carotid surgery) within the previous 30 days or planned in the next 90 days after enrollment; Indication for warfarin or heparin beyond enrollment; Pregnancy or unwilling to use contraception for the duration of this study; Severe condition that would prevent participation in study procedures |
| ***Study Design*** | A multicenter, randomized, open-label, outcome assessor-blinded trial comparing medical therapy alone with medical therapy plus stenting in patients with TIA or non-disabling ischemic stroke with 70% to 99% stenosis of a major intracranial artery |
| ***Primary Outcomes*** | Stroke or death within 30 days after enrollment; Stroke in the territory of qualifying artery beyond 30 days through 12 months |
| ***Secondary And Safety Outcomes*** | Disabling stroke or death within 3 years; Stroke in the same territory within 2 years; Stroke in the same territory within 3 years; Any stroke, severe TIA, cardiovascular events related to stenting or medical therapy within 3 years; Death within 3 years; Symptomatic intracranial hemorrhage, or death within 1 year; Causes of death within 3 years |

**Figure S1 Sensitivity analysis of the data with heterogeneity greater than 50%: any stroke or death within 1 year**


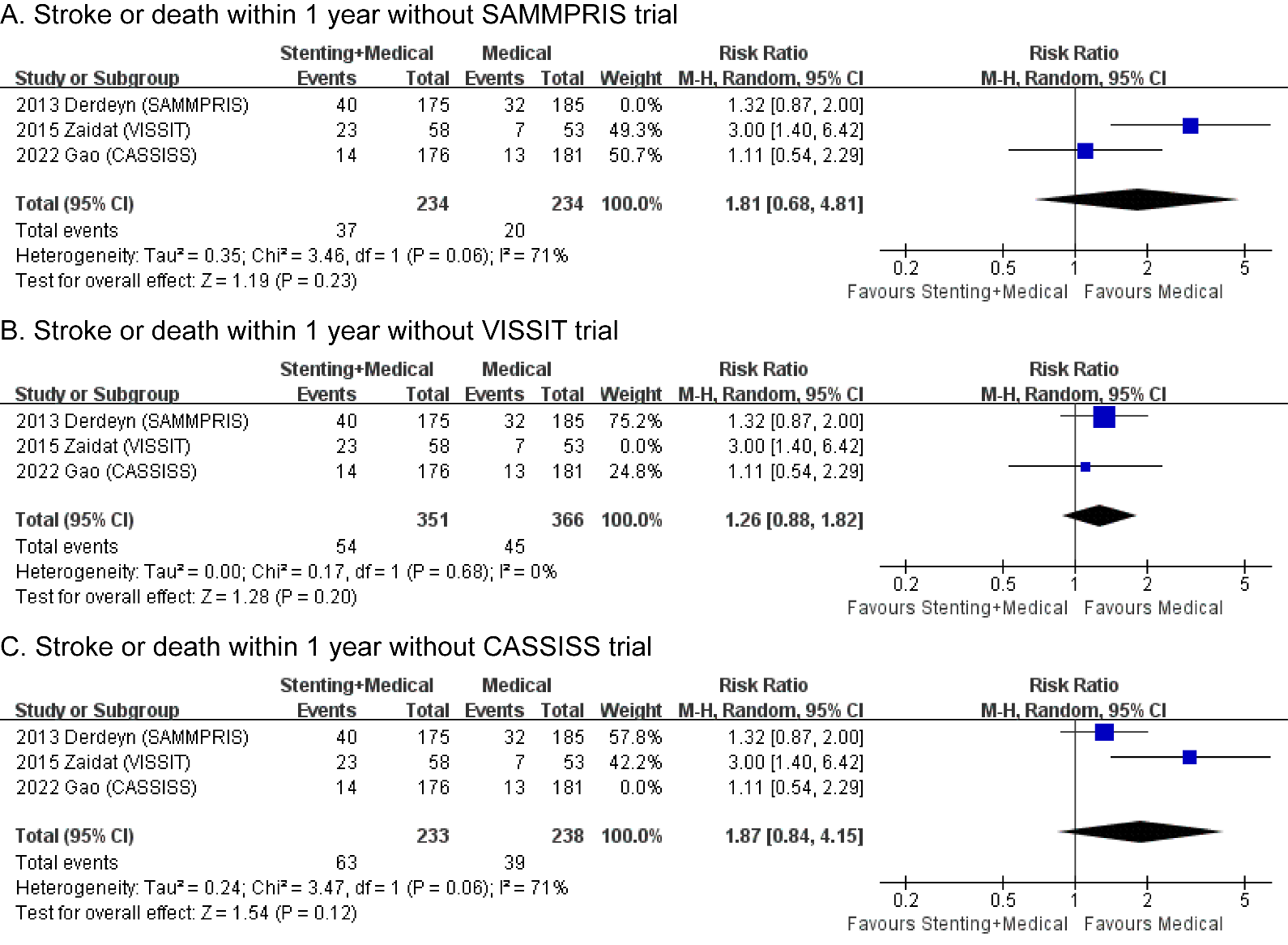


**Figure S2 Sensitivity analysis of the data with heterogeneity greater than 50%: any ischemic stroke within 1 year**

**
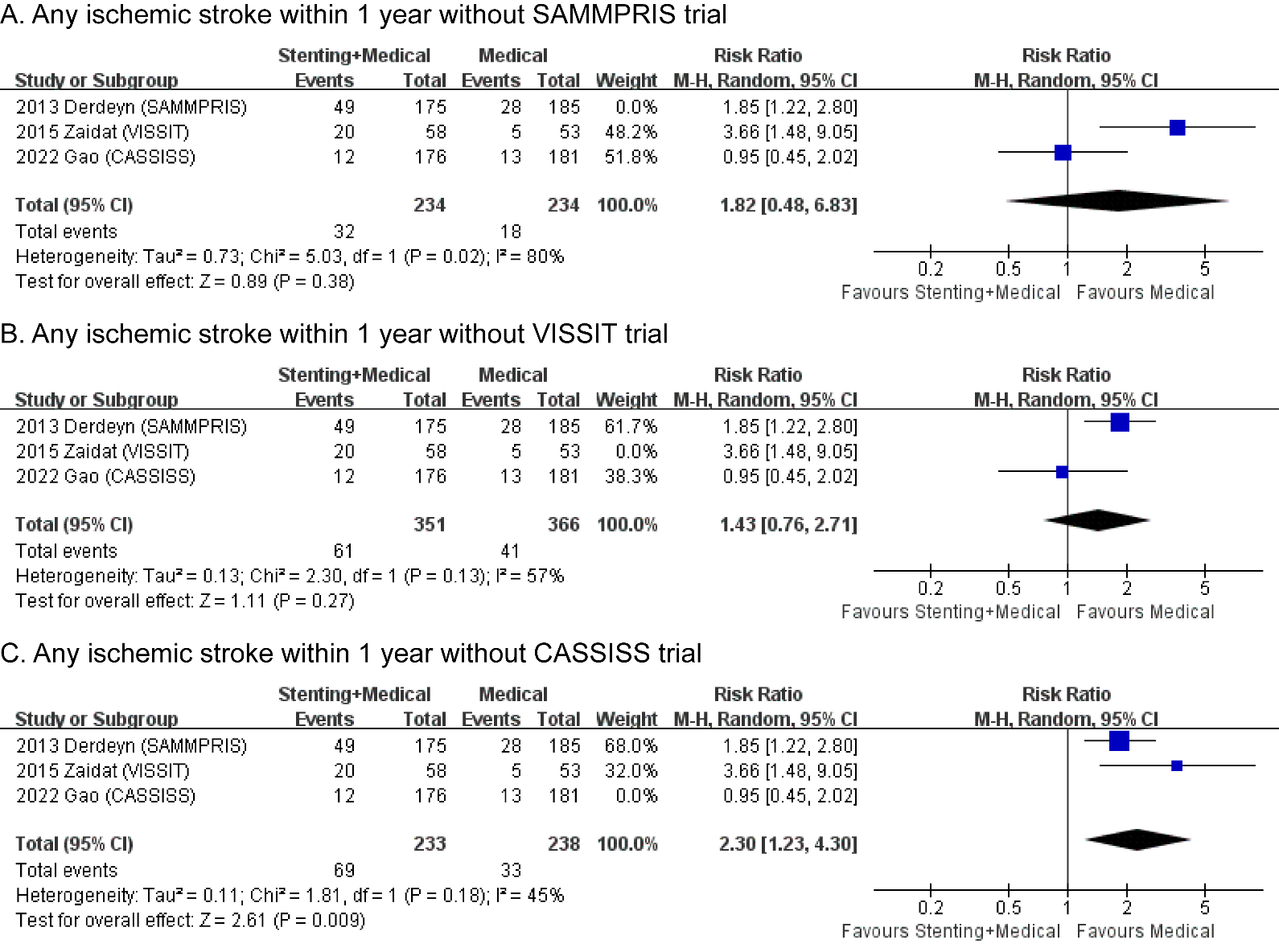
**

**Figure S3 Sub-analysis of time from qualifying event to randomization in primary outcome: stroke and death within 30 days.**


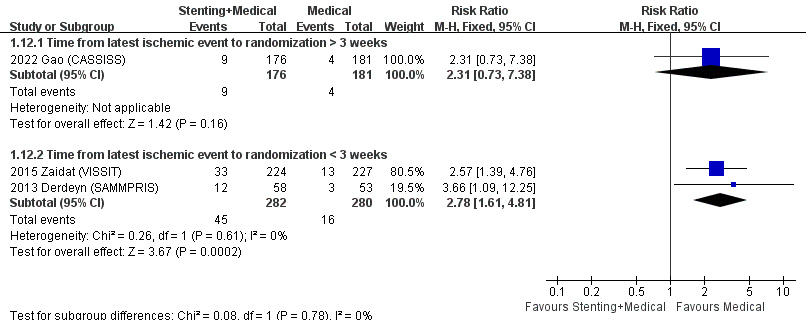


**Figure S4 Sub-analysis of time from qualifying event to randomization in primary outcome: stroke and death within 1 year.**

**
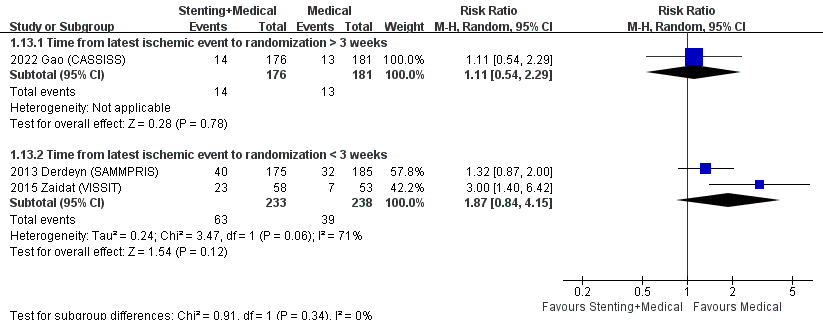
**

**Figure S5 Sub-analysis of stent type in primary outcome: stroke and death within 30 days.**


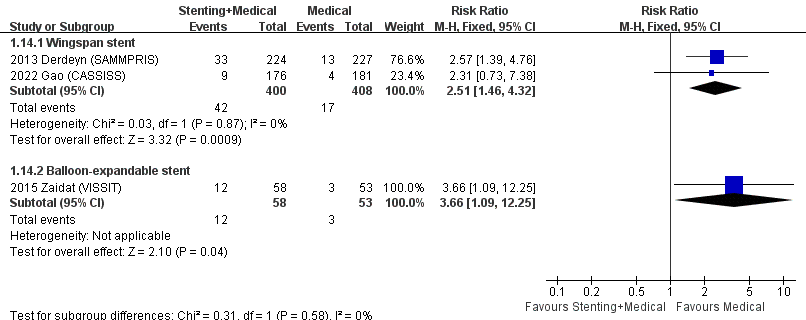


**Figure S6 Sub-analysis of stent type in primary outcome: stroke and death within 1 year.**

**
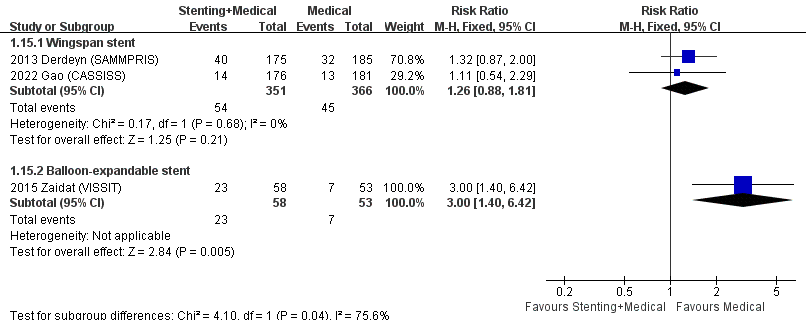
**
